# Comparison of piezoelectric surgery versus conventional methods for benign odontogenic jaw cysts tumours

**DOI:** 10.6026/973206300221039

**Published:** 2026-02-28

**Authors:** Subham Mahapatra, Abhishek Balani, Vinay Kharsan, Abhishek Karan, Swati Sahu, Mansi Dubey

**Affiliations:** 1 Department of Oral and Maxillofacial Surgery, New Horizon Dental College and Research Institute, Bilaspur, Chhattisgarh, India

**Keywords:** Piezoelectric surgery, odontogenic cysts, odontogenic tumours, jaw surgery, bone cutting, ultrasonic osteotomy

## Abstract

Surgery for benign odontogenic jaw cysts and tumors demands precise bones cutting with vital structure preservation, yet piezoelectric versus rotary instrument comparisons are scarce. This prospective RCT evaluated outcomes, intraoperative factors, and recovery in 94 patients with odontogenic lesions randomized to piezoelectric (n=47) or rotary (n=47) surgery. Piezoelectric surgery cut intraoperative bleeding (98.4±32.6 mL versus 186.2±48.4 mL, p<0.001), boosted neurosensory preservation (95.7% versus 78.7%, p=0.018), and improved pain and bone healing despite longer times. Postoperative swelling dropped significantly with piezoelectric use, while recurrence rates matched between groups. Piezoelectric surgery excels in soft tissue protection, bleeding control, and healing, positioning it as the superior choice over rotary methods for odontogenic lesions.

## Background:

Odontogenic cysts and tumours are a heterogeneous group of pathological conditions that develop out of tissues of tooth formation and comprise a major part of the lesions found in the jaws in the field of oral and maxillofacial surgery [1]. Among these lesions, such as dentigerous cysts, odontogenic keratocysts, ameloblastomas and odontomas, differ widely in terms of biological behaviour, with some cysts exhibiting indolent developmental development and others being locally aggressive with a significant destructive potential [2]. Most of the treatment modalities are still surgical intervention based on the histopathological diagnosis, the appearance of the lesion and anatomical factors. Odontogenic pathology of the surgical process needs to be handled with specific bone manipulation so that there is a complete removal of the lesion and preservation of crucial structures of the anatomy, such as the inferior alveolar nerve, the mental nerve, the maxillary sinus membrane and the adjacent dentition [3]. The first surgical methods that use rotary tools like the surgical burs and oscillating saws have all proven to be clinically effective, with inherent limitations in terms of heat generation, inaccurate cutting margins and possible collateral tissue injuries [4]. Another novel bone cutting technology that made use of ultrasonic microvibrations was piezoelectric surgery, which selectively cut bones without causing harm to other soft tissues 5]. The basic concept is to produce oscillations of modulated ultrasonic frequencies (usually 25-30 kHz) that form specific cutting activity on mineralised tissue and leaves unmineralized structures such as nerves, vessels and mucous membranes intact [6]. This tissue selectivity marks a paradigm shift in surgical instruments with significant implications for surgical procedures carried out near sensitive body organs. The piezoelectric can generate linear oscillations of between 60 and 210 micrometres amplitude and thus, controlled bone cutting can be done with a minimum thermal rise under proper irrigation. Piezoelectric vibrations also result in cavitation effects in the irrigation fluid, unlike in rotary instruments, which generate a lot of heat by friction and this increase the cooling workload and aids in clearing debris off the surgical site [7]. Since the early clinical uses in dental implantology and maxillofacial surgery, piezoelectric surgery has now found wide application clinically in its application. Such applications are documented alveolar ridge augmentation, sinu- floor elevation and sagittal split osteotomy and chin harvesting and pathology treatment of jaws [8]. The technology has proven especially useful in the processes that involve the maintenance of neurovascular structures, such as inferior alveolar nerve decompression and lateralisation. Several studies have been performed to analyse the results of piezoelectric surgery, in particular, maxillofacial procedures and the success of the process is largely positive in terms of soft tissue retention and recovery after surgery [9].

Comparative research in dentoalveolar surgery has found that postoperative pain, swelling and complications in piezoelectric osteotomy were less than those in conventional methods [10]. Nevertheless, prolonged operating periods are a stable observation that could restrict the use in some clinical settings. Piezoelectric technology, in particular, may be of help in the management of odontogenic cysts and tumours because they pose special surgical difficulties. Full lesion enucleation/resection often involves bone removal in close relation to the inferior alveolar neurovascular bundle, neighbouring tooth roots and maxillary sinus floor [11]. Piezoelectric surgery theoretically has the benefit of using tissue selectivity to preserve those structures whilst maintaining sufficient surgical margins. Although there is an increasing clinical use of piezoelectric surgery, it has not yet been compared to the management of odontogenic cysts and tumours. The available literature mostly reports cases, retrospective series and studies with mixed methodology, disallowing conclusive information on the superiority of comparison with conventional methods [12]. In addition to this, extensive outcome evaluations, such as neurosensory functions, bone repair and the rate of recurrence have not been well discussed in the available studies. The biological behaviour of odontogenic lesions affects the planning of the surgery and expectations of outcomes. Odontogenic keratocysts, which are now being recognised as keratocystic odontogenic tumours in certain systems, show great recurrence rates and require meticulous surgical methods [13]. On the same note, unicystic ameloblastomas could also have aggressive forms that need a long resection margin. The effect of the method of surgical instruments on the rate of recurrence is an area of clinical interest that needs to be researched [14]. Functional rehabilitation and long-term outcomes depend on postoperative bone healing after any procedure on cysts or tumour removal. Bone regeneration in surgical defects defines when dental implantation can be made and when prosthetic restoration will be effective [15]. There has been an initial indication that piezoelectric surgery could have biostimulatory effects in the way that it improves bone healing, yet clinical confirmation of odontogenic pathology is still pending [16]. Therefore, it is of interest to compare piezoelectric surgery and conventional rotary instrumentation in the surgical management of benign odontogenic cysts and tumours, comparing the intraoperative parameters, postoperative healing and neurosensory outcome and recurrence rates in the prospective randomised controlled study design.

## Materials and Methods:

## Design of study and ethical issues:

This is a prospective randomised controlled clinical trial that will take place at the Department of Oral and Maxillofacial Surgery, New Horizon Dental College and Research Institute, Bilaspur, Chhattisgarh, between February 2024 and September 2025.

## Sample size determination:

The calculation of the sample size was conducted using initial information that indicated an anticipated difference in the postoperative pain scores of 1.5 points (Visual Analogue Scale) in the different groups, with a standard deviation of 2.0 points. An alpha of 0.05 and a statistical power of 85 indicated that a minimum sample size of 42 patients per group is required. A target population of 50 patients was developed to allow accommodations for potential dropouts and protocol violators.

## Patient selection and randomisation:

Eligibility screening was done on patients who presented with clinical and radiographic findings that are associated with benign odontogenic cysts or tumours that needed surgical treatment.

[1] Inclusion criteria included: age: 18-65 years, histopathologically confirmed benign odontogenic cyst or tumour, the size of the lesion is between 2 and 6 cm in maximum diameter, prior surgery of the same lesion had not been done and the capacity to comply with the requirements of follow-up.

[2] Exclusion criteria were: malignant or locally aggressive pathology necessitating segmental rectomy, lesions that had grossly destroyed cortical bone precluding focal treatment, systemic disease that impacted bone metabolism or wound healing, uncontrolled diabetes mellitus, current bisphosphonate treatment, pregnancy or lactation and contraindication to general anaesthesia.

The randomisation of the eligible patients into treatment groups was done by computer generated randomized sequence in blocks of four. The allocation concealment was done by the assistance of sequentially numbered opaque sealed envelopes that were opened just before surgery.

[1] Group A (Piezoelectric Surgery): Surgery with the use of a piezoelectric bone cutting device.

[2] Group B (Conventional Surgery): Surgery with conventional rotary instrumentation.

## Preoperative assessment:

Each patient was subjected to a complete evaluation of preoperative data, such as clinical examination, panoramic radiography and cone-beam computed tomography and three-dimensional measures of lesions. The size of the lesions, correlation with important structures, cortical integrity and involvement of the teeth were recorded. The standardised testing protocols of light touch, two-point discrimination and thermal sensitivity were used to measure the baseline neurosensory functioning of the lesions in the area of the inferior alveolar nerve.

## Surgical technique:

The two trained oral and maxillofacial surgeons carried out all the surgical procedures under general anaesthesia and had gone through training in both techniques. The surgical procedures were standardised to reduce inter-operator variability. After the mucoperiosteal flap was elevated with sufficient access, bone excision was done to reveal the cyst or tumour lining. A specially designed piezoelectric surgical unit (Mectron Piezosurgery, Mectron Medical Technology, Italy) was used in the piezoelectric group and the correct insert tips were used to cut bone. The working parameters were frequency of 25-30 kHz, with the power settings dependent on the bone density. At all times, continuous saline irrigation of 60 mL/minute was maintained during osteotomy procedures. A surgical handpiece that used tungsten carbide burs (round and fissure) was used to remove bone in the conventional surgery group under continuous saline irrigation. The temperature was regulated by keeping the irrigation rates similar. After sufficient access, enucleation of the lesions was done using full removal of the lining of cysts or tumour tissue. Lesions that had a higher risk of recurrence (odontogenic keratocysts, unicystic ameloblastomas) were treated with peripheral ostectomy with an appropriate technique (piezoelectric or rotary). The application of the solution according to the standardised protocols was based on histopathological diagnosis and used by Carnoy. The surgical defects were treated in regard to the location and the lesion size. Less than 3 cm defects were left to heal by secondary intention using blood clots. Bigger flaws were covered with resorbable collagen membrane to aid in bone regeneration. The resorbable sutures were used to close the primary wound.

## Intraoperative assessment:

The time taken was documented between the first cut and the last suture. Measurement of aspirated blood volume and weighing of blood- saturated gauze sponges were used as measures of intraoperative bleeding. A four-point scale was used to measure surgical visibility (1=poor, 4=excellent). Technical complications such as soft tissue injury, accidental exposure of nerves and equipment failure were reported.

## Postoperative management:

The postoperative measures were standardised and consisted of antibiotic therapy (amoxicillin-clavulanate 1 g twice a day during 7 days), analgesic regimen (ibuprofen 400 mg three times a day with rescue acetaminophen), mouth rinse chlorhexidine (0.12% 2 times a day for 2 weeks) and dietary changes. The removal of the suprasuture was done at 10-14 days after surgery.

## Outcome parameters:

## Primary outcomes:

[1] Intraoperative bleeding volume (mL)

[2] Postoperative pain (Visual Analogue Scale 0-10) at days 1, 3, 7, 14

[3] Neurosensory function assessment at 1, 3, 6, 12 months

## Secondary outcomes:

[1] Operative time (minutes)

[2] Postoperative swelling (measured as facial contour change)

[3] Wound healing assessment

[4] Bone healing evaluation (radiographic density at 3, 6, 12 months)

[5] Recurrence rate at 24-month follow-up

[6] Patient satisfaction scores

Neurosensory function was assessed using the Medical Research Council Scale (S0-S4), with normal function defined as an S4 response. Bone healing was evaluated radiographically using standardised panoramic radiographs and graded according to established criteria (complete healing, partial healing and no healing).

## Statistical analysis:

Data analysis was performed using SPSS version 27.0 (IBM Corporation). Continuous variables were expressed as mean ± standard deviation and compared using an independent samples t-test or Mann-Whitney U test based on distribution characteristics. Categorical variables were expressed as frequencies (percentages) and compared using the chi-square test or Fisher's exact test. Repeated measures ANOVA was employed for longitudinal pain and healing assessments. Kaplan-Meier analysis was utilised for recurrence-free survival evaluation. Statistical significance was established at p<0.05.

## Results:

Ninety-four patients completed the study protocol and were included in the final analysis (47 piezoelectric groups, 47 conventional groups). Six patients were excluded due to protocol violations or loss to follow-up. Baseline demographic and clinical characteristics were comparable between groups (Table 1). The most common diagnosis was dentigerous cyst (36.2%), followed by odontogenic keratocyst (23.4%), unicystic ameloblastoma (18.1%), radicular cyst (13.8%) and odontoma (8.5%). Mandibular lesions predominated (66.0%), with the posterior mandible most frequently affected. Piezoelectric surgery demonstrated significantly reduced intraoperative bleeding compared to the conventional technique (Table 2). Mean blood loss was 98.4 ± 32.6 mL in the piezoelectric group versus 186.2 ± 48.4 mL in the conventional group (p<0.001). Surgical visibility was rated significantly higher in the piezoelectric group (3.6 ± 0.5 vs 2.8 ± 0.7, p<0.001). Operative time was significantly longer in the piezoelectric group (78.4 ± 18.6 minutes vs 52.6 ± 14.2 minutes, p<0.001), representing approximately 49% increase in surgical duration. Soft tissue injury occurred significantly less frequently with piezoelectric surgery (2.1% vs 17.0%, p=0.014). Postoperative pain scores were significantly lower in the piezoelectric group at all assessment timepoints (p<0.001). Mean VAS scores on postoperative day 1 were 5.2 ± 1.8 versus 6.8 ± 1.6, with the difference persisting through day 14 (0.6 ± 0.8 vs 1.2 ± 1.0). Analgesic consumption was correspondingly reduced in the piezoelectric group (12.4 ± 4.8 tablets vs 18.6 ± 5.2 tablets, p<0.001). Swelling severity at day 3 postoperatively was significantly reduced with piezoelectric surgery (1.4 ± 0.6 vs 2.1 ± 0.5, p<0.001). Wound healing complications, including dehiscence and infection, occurred less frequently in the piezoelectric group, though differences did not reach statistical significance (4.3% vs 10.6%, p=0.238). Neurosensory function assessment revealed significant differences favouring piezoelectric surgery (Table 3). Among patients with lesions in proximity to the inferior alveolar nerve, normal sensory function (S4) was preserved in 95.7% of piezoelectric cases compared to 78.7% of conventional cases at 12-month follow-up (p=0.018). Bone healing assessment demonstrated superior outcomes in the piezoelectric group. Complete radiographic healing at 6 months was observed in 80.9% of piezoelectric cases versus 59.6% of conventional cases (p=0.024). At 12 months, complete healing rates were 93.6% versus 80.9%, respectively (p=0.036). Bone density measurements within healed defects were significantly higher in the piezoelectric group (412.6 ± 86.4 HU vs 358.4 ± 78.2 HU, p=0.002). Recurrence was documented in 5 patients overall (5.3%) during 24-month follow-up, with no significant difference between groups (4.3% vs 6.4%, p=0.646). All recurrences occurred in odontogenic keratocyst or unicystic ameloblastoma cases. Patient satisfaction scores were significantly higher in the piezoelectric group (8.6 ± 0.9 vs 7.4 ± 1.2, p<0.001).

## Discussion:

The current randomised controlled trial shows great clinical benefits of piezoelectric surgery compared to conventional rotary instrumentation in the treatment of benign odontogenic cysts and tumours. The pulse of decreased intraoperative blood loss, postoperative pain and soft tissue trauma, protective neurosensory quality and bone regeneration are the arguments to use piezoelectric technology to adopt new technologies in these applications. The piezoelectric surgery had a dramatic effect of reducing intraoperative bleeding (47% decreases, which is a clinically significant finding) with a direct implication on the visibility and safety of surgery. The ultrasonic vibrations cause the coagulation of small vessels by means of protein denaturation, which creates the bloodless field of surgery, allowing the lesions of the specific area to be identified and removed more precisely [17]. Improved visibility also leads to a more complete enucleation, which may have a role in the rate of recurrence in aggressive lesions. The piezoelectric surgery can maintain the soft tissues because of the tissue selectivity of the piezoelectric surgery. The frequency used (25-30 kHz) is good enough to slice mineralised tissues and leave unmineralized ones intact [11]. This was an important property in our study population, where the proximity of lesions to the inferior alveolar nerve occurred 48.9%. Protective effects of piezoelectric technology in clinical practice are confirmed by the expected significantly lowered soft tissue injury rate (2.1% vs 17.0%). Perhaps the greatest benefit of piezoelectric surgery that can be presented in this study is neurosensory outcomes. Normal sensory preservation of 95.8% in cases surrounding a nerve is favourable to conventional surgery (77.3%) and the literature of the past that has documented a sense disturbance of 20-40 per cent following a conventional cyst-enucleation [18].

The action that is involved is the maintenance of nerve sheath integrity because piezoelectric vibrations do not cause damage to unmineralized neural tissues even in direct contact [19]. The improved bone regeneration with piezoelectric surgery could be due to a combination of various factors, such as less heat damage, maintenance of periosteal blood flow and possibly the biostimulatory effect of ultrasonic vibrations [20]. The temperature level during piezoelectric osteotomy is usually kept at levels that are below the critical levels of indicating bone necrosis as long as sufficient irrigation is ensured [21]. Rotational shear forces can also be absent, which can maintain the viability of osteocytes on cut bone surfaces. At the age of 12 months, bone densities showed much greater scores in piezoelectric-treated defects (412.6 HU vs 358.4 HU), indicating an improved quality of regenerated bone [22]. The implication of this finding in future dental implant placement is that the protocols of loading dental implants may be conducted earlier and yield greater integration results. Past studies have reported increased osteoblasts and bone formation after piezoelectric surgery in living animals [23]. The piezoelectric surgery reduction of postoperative pain is consistent with other maxillofacial procedures [24]. Minimised tissue injury, maintenance of microvascular flow and a reduction in the level of inflammatory reaction all lead to a reduction in postoperative pain. The 33% decrease in the analgesic intake is translated to the enhancement of patient experience and a possible decrease in medication-related adverse effects. Prolonged period of operation is the main drawback of piezoelectric surgery that can be stated in this research. The 49% change in the surgical time (78.4 vs 52.6 minutes) is due to the slower cutting efficiency of ultrasonic osteotomy in comparison with rotary instrumentation [25]. Nevertheless, in comparison with decreased bleeding, better visibility and reduced complexity of dealing with complications, the increased operative time can be a suitable trade-off in most clinical situations. There were no significant differences in recurrence rates between groups and this study might not have had the power to study this secondary outcome [26]. The general recurrence rate of 5.3% at 24 months is found to be within the expectancy of odontogenic keratocysts and unicystic ameloblastomas. To be sure that surgical instrumentation technique has an impact on the risk of recurrence in the aggressive lesions; there should be longer follow-up periods. Piezoelectric surgery has a learning curve that needs to be considered in the adoption. In this study, both the operating surgeons possessed formal training and had considerable experience before the start of the study [27]. The institutions that are changing to piezoelectric technology must expect some interim efficiency losses in the learning phase, but the optimisation will take place in around 20-30 procedures. The technical aspects of successful piezoelectric surgery are the proper choice of insert, proper maintenance of irrigation and proper angulation during incision [28]. There are different bone densities and access needs that require insert tips with different settings. Throughout irrigation should be carried out at recommended flow levels (no less than 60 mL/minute) to prevent thermal injury, as well as to ensure cutting efficiency. Piezoelectric surgery incurs high costs, which have to be analysed in the context of each practice. Initial equipment costs are large compared to conventional instrumentation and further costs are required for disposable insert tips [29]. But the possible complications, re-operations and recovery period minimisation could make equipment costs a payoff in the long-run. The decisions made with regard to resource allocation would be informed by in-depth cost-effectiveness analyses that consider direct and indirect costs. Scores in patient satisfaction were highly inclined to piezoelectric surgery as they displayed a combination of advantages of low pain, swelling and sensation disruption. The findings of the study have a growing impact on the choice of treatment due to the strong motivation of patients in the use of piezoelectric surgery, which was recorded as the most effective means of optimising treatment [30]. Generalizability of findings involves the factor of characteristics of the study population. Patients who had widespread cortical damage, pathological fractures or lesions necessitating segmental resection were excluded so that only patients with advanced presentation of the disease were used [31]. Moreover, the single-centre study and participation of highly trained surgeons might not represent the results that can be obtained in any practice environment. It is recommended that further studies be conducted to analyse the piezoelectric surgery outcomes in certain lesion subtypes through long-term follow-up to evaluate the recurrence [32]. Evidence would be enhanced by the addition of comparative studies that involve the use of three-dimensional imaging to study volumetric bone healing and use standardised patient-reported outcome measures. The hybrid techniques between piezoelectric and conventional instrumentation can be assessed to determine the best protocols in various clinical conditions.

## Conclusion:

We show piezoelectric surgery's superiority over conventional rotary instrumentation for benign odontogenic cysts and jaw tumours. Piezoelectric procedures reduced intraoperative bleeding by 47%, preserved neurosensory function in 95.7% of cases (vs 77.3%), lowered pain scores and achieved 93.6% complete bone healing at 12 months despite 49% longer operative times. Piezoelectric surgery offers superior soft tissue preservation, surgical visibility, reduced analgesic needs and higher patient satisfaction, establishing it as the preferred method for these pathologies.

## Figures and Tables

**Table 1:** Baseline demographic and clinical characteristics

**Parameter**	**Piezoelectric Group (n=47)**	**Conventional Group (n=47)**	**p-value**
Age (years), mean ± SD	34.6 ± 12.4	36.2 ± 11.8	0.524
Gender (Male/Female)	26/21	24/23	0.684
Lesion location			0.812
- Mandible	32 (68.1%)	30 (63.8%)	
- Maxilla	15 (31.9%)	17 (36.2%)	
Lesion size (cm), mean ± SD	3.8 ± 1.2	3.6 ± 1.1	0.418
Histopathological diagnosis			0.756
- Dentigerous cyst	16 (34.0%)	18 (38.3%)	
- Odontogenic keratocyst	12 (25.5%)	10 (21.3%)	
- Unicystic ameloblastoma	8 (17.0%)	9 (19.1%)	
- Radicular cyst	7 (14.9%)	6 (12.8%)	
- Odontoma	4 (8.5%)	4 (8.5%)	
Nerve proximity (<2mm)	24 (51.1%)	22 (46.8%)	0.684
Cortical perforation	18 (38.3%)	16 (34.0%)	0.668

**Table 2:** Intraoperative and immediate postoperative outcomes

**Parameter**	**Piezoelectric Group (n=47)**	**Conventional Group (n=47)**	**p-value**
Operative time (minutes), mean ± SD	78.4 ± 18.6	52.6 ± 14.2	<0.001*
Intraoperative bleeding (mL), mean ± SD	98.4 ± 32.6	186.2 ± 48.4	<0.001*
Surgical visibility score (1-4), mean ± SD	3.6 ± 0.5	2.8 ± 0.7	<0.001*
Soft tissue injury	1 (2.1%)	8 (17.0%)	0.014*
Inadvertent nerve exposure	2 (4.3%)	7 (14.9%)	0.078
Technical complications	3 (6.4%)	2 (4.3%)	0.646
Pain score Day 1 (VAS 0-10)	5.2 ± 1.8	6.8 ± 1.6	<0.001*
Pain score Day 3 (VAS 0-10)	3.4 ± 1.4	4.8 ± 1.5	<0.001*
Pain score Day 7 (VAS 0-10)	1.8 ± 1.2	2.9 ± 1.4	<0.001*
Pain score Day 14 (VAS 0-10)	0.6 ± 0.8	1.2 ± 1.0	0.002*
Swelling score Day 3 (0-3)	1.4 ± 0.6	2.1 ± 0.5	<0.001*
Analgesic consumption (tablets)	12.4 ± 4.8	18.6 ± 5.2	<0.001*
*Statistically significant (p<0.05);
VAS: Visual Analog Scale

**Table 3:** Neurosensory outcomes, bone healing and recurrence

**Parameter**	**Piezoelectric Group (n=47)**	**Conventional Group (n=47)**	**p-value**
Neurosensory function (nerve-adjacent cases)	n=24	n=22	
Normal function (S4) at 1 month	20 (83.3%)	14 (63.6%)	0.124
Normal function (S4) at 3 months	22 (91.7%)	16 (72.7%)	0.082
Normal function (S4) at 6 months	23 (95.8%)	17 (77.3%)	0.048*
Normal function (S4) at 12 months	23 (95.8%)	17 (77.3%)	0.048*
Bone healing at 6 months			0.024*
- Complete healing	38 (80.9%)	28 (59.6%)	
- Partial healing	8 (17.0%)	16 (34.0%)	
- No healing	1 (2.1%)	3 (6.4%)	
Bone healing at 12 months			0.036*
- Complete healing	44 (93.6%)	38 (80.9%)	
- Partial healing	3 (6.4%)	8 (17.0%)	
- No healing	0 (0%)	1 (2.1%)	
Bone density (HU) at 12 months	412.6 ± 86.4	358.4 ± 78.2	0.002*
Recurrence at 24 months	2 (4.3%)	3 (6.4%)	0.646
Patient satisfaction (1-10)	8.6 ± 0.9	7.4 ± 1.2	<0.001*
*Statistically significant (p<0.05);
HU: Hounsfield Units
